# A Pocket Atlas of the Descriptive Anatomy of the Human Body

**Published:** 1846-11

**Authors:** 


ART. VII.—*A Pocket Atlas of the Descriptive Anatomy of the Human Body.* By J. N. MASSE, Professor of Anatomy, Paris. Translated from the last Paris edition and edited by GRANVILLE SHARP PATTISON, M.D., Professor of Anatomy in the University of New York; Member of the Medico-Chirurgical Society of London; of the Wernerian Society of Natural History of Edinburgh; of the Société Medicale D'Emulation, and Société Philomatique of Paris. New York: Harper & Brothers, publishers, 82 Cliff street. 1845.

Anatomy, confessedly, is at the basis of Medical Science, and all true helps to the attainment of a thorough knowledge of it will be cordially welcomed by the profession. That such is the character of the little work the title of which is given above, is the conviction of a veteran anatomist and medical teacher, whose long experience in that department entitles his opinions to great weight. Professor Pattison shall speak for himself touching the merits of the volume which has just appeared under his auspices.

"The editor feels much gratification in being enabled to present to the profession of the United States an American edition of the beautiful "Pocket Anatomical Atlas" of Professor J. N. Masse, of Paris. As a teacher of Anatomy, he has long felt the want of such a work to recommend to his pupils; and, recalling to his mind the difficulties he encountered himself, as a student, in the cultivation of anatomical science, he is enabled to form a just estimate of the value of such an atlas. There are, it is true, already in existence many valuable anatomical atlases. These are, however, not only too voluminous and too expensive to supply the wants of the great body of the profession, but, as it has been observed by the author, from the different parts of the same organ being represented in different engravings, the student, from his being obliged, in consulting them, to refer to different plates, becomes confused, and is unable to form a correct idea of the true structure of the organ which he studies. Again, from their size, they are only suited for consultation at home; they cannot be carried into the anatomical theatre and the dissecting room, which, in the opinion of the editor, is a great objection against their use. The pocket form of the Anatomical Atlas of Professor Masse enables the student to make it his constant companion. He places it before his eye as he listens to the demonstration of the professor in the lecture-room; and on his return home on his examining the plates, the subjects which he has heard demonstrated, the lesson taught by his teacher is forcibly recalled to his mind. In the dissecting-room, the Pocket Atlas serves as a guide to the student in the prosecution of his dissections; it not only teaches him how to expose the organs, the structure of which he is investigating, but the engravings so clearly exhibit them to his eye, and the text furnishes such satisfactory information as to the name and locality of every part of the structure he has unveiled, that the necessity for the assistance of a demonstrator is, in a great measure, obviated. To the physician or surgeon who has no means of referring to the *subject*, Masse's Atlas will be found an invaluable work for reference. When the former wishes to refer to the position of any particular viscus, and to study its relations, or when symptoms depending on nervous connexion arise in disease which he cannot explain, referring to this Atlas, all his difficulties are removed. When the latter prepares himself for the performance of a surgical operation, and when, in vain, he attempts to recall to his mind surgical relations long since seen, and now only confusedly remembered, referring to his Atlas, the vision of the past is recalled to the mind in all its original freshness, and with confidence he is enabled to perform his operation. Such being the fact, invaluable as the possession of this Pocket Atlas is to the medical student, it is, if possible, still more valuable to the country physician and surgeon; and, from the very low price at which it is furnished, we are much mistaken if it does not obtain a more extensive circulation than any similar work ever before published in the United States.

This work, together with all the others noticed in the foregoing pages, may be had at the book stores of Messrs. Maxwell and Prescott in this city. We should suppose students would do well to supply themselves with the Atlas, which must greatly facilitate the business of unraveling the complicated structure of the human body.

